# Management of a Periodontally Hopeless Tooth with Intentional Re-implantation

**DOI:** 10.4317/jced.61873

**Published:** 2025-07-01

**Authors:** Divya Konduri, Jagadish Reddy Gooty, Vikram Reddy Guntakandla, S. Jaya Prada Reddy, Raja Babu Palaparthy, Juliet Josephin

**Affiliations:** 1 Post Graduate Student, Department of Periodontology, Kamineni Institute of Dental Sciences, Narketpally, Telangana-508254; 2 Professor, Department of Periodontology, Kamineni Institute of Dental Sciences, Narketpally, Telangana; 3 Professor and Head, Department of Conservative and Endodontics, Kamineni Institute of Dental Sciences, Narketpally, Telangana; 4 Professor and Head, Department of Periodontology, Kamineni Institute of Dental Sciences, Narketpally, Telangana; 5 Reader, Department of Periodontology, Kamineni Institute of Dental Sciences, Narketpally, Telangana

## Abstract

Intentional replantation is a process that involves purposeful tooth extraction and subsequent reinsertion of the removed tooth. Intentional replantation may be a therapeutic option worth considering to preserve the natural teeth and prevent tooth extraction, even though the success rate is not always high. Systemically healthy male patient was referred to our department. Diagnosis was chronic periodontitis and class III mobility was noted at the right maxillary central incisor with complete periodontal attachment loss. After phase I therapy periodontally involved tooth was extracted, endodontic treatment was accomplished, the tooth was replanted and stabilized with a composite wire splint. At the end of 3 years tooth was in function with alveolar bone gain. Intentional replantation provided long-term maintenance of patient’s own teeth and can be considered as an alternative treatment option tooth with hopeless prognosis.

** Key words:**Ankylosis, Chronic Periodontitis, Periodontal Attachment Loss, Root Resorption, Tooth Replantation, Tooth Extraction.

## Introduction

The primary goal of periodontal therapy is to repair the teeth. However, addressing extensive periodontal damage in anterior teeth is challenging on both biological and functional levels. In cases of severe periodontal disease, both surgical and non-surgical treatment options often prove ineffective, necessitating tooth extraction (1).

Intentional replantation is defined as intentional removal of tooth and reinsertion into the ex-traction socket before or after proper endodontic treatment (2). It entails repositioning the extracted tooth, root-end resection/filling, and atraumatic extraction of the problematic tooth (3). To some authors, deliberate replantation is considered a desperate attempt to preserve a lost tooth for unrelated causes (3,4). An approach for treating extensive periodontal damage of the anterior teeth is intentional replantation.

Intentional replacement of teeth with moderate-to-severe periodontal disease is generally considered unsuiTable, and a healthy periodontal state is a precondition for long-term success (5). While the majority of authors stated that periodontal involvement precluded replantation, other studies found that periodontally affected teeth could benefit from replantation (6,7). Following an endodontically mistreated and periodontally affected mandibular first premolar, Lu (6) transplanted the tooth, which remained asymptotically functional for 32 months. Intentional replantation of periodontally compromised teeth was carried out by Baykara & Eratalay (7) who monitored the patients for eight years. Results have shown that the patient survived with healthy gingiva, a significant reduction in pocket depth, signs of newly formed bone, and ankylosis (7).

The main advantage of this technique is that tooth surfaces, including inaccessible areas, can be fully visualized and used without damaging adjacent periodontal tissues and contributes to the reconstruction of healthy periradicular tissues (8,9). Indications for intentional replantation are limited; however, in an attempt to preserve natural dentistry, it is truly a treatment option if more traditional forms of treatment fail or are impossible. Low cost and takes less time are advantages of this procedure. However, there is always a risk of root fracture and root resorption may occur over a period of time (10).

The aim of this case report was to demonstrate the clinical and radiographic three-year results of intentional replantation of periodontally involved tooth.

## Case Report

**Figure 1:**
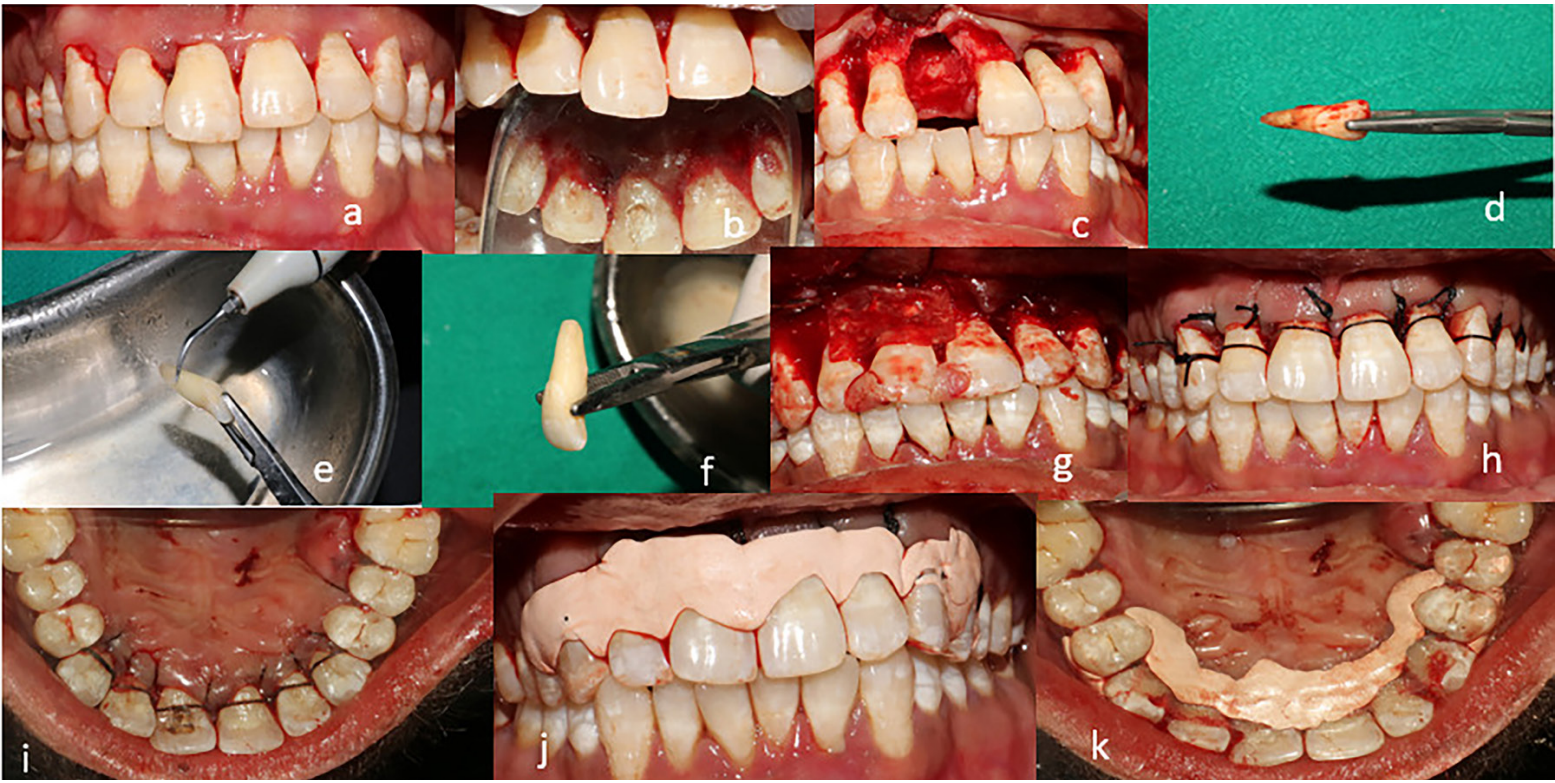
a,b) pre-operative images c) Atraumatic extraction of hopeless tooth d) Extracted tooth e) Removal of calculus and necrotic cementum f) After root planing g) Periodontally involved sites were thoroughly debrided, G-graft and GTR membrane were placed h,i) Sling sutures were placed j,k) Periodontal pack was placed.

In this 28-year-old individual with a normal systemic state of health, radiographs and clinical results revealed localized Periodontitis. On the left and right central incisors, a combination of significant periodontal bone loss, deep periodontal pockets, pathological migration, suppuration, bleeding on probing, sensitivity on the upper right central and gingival recession was detected. Attachment loss was moderate to severe, and oral hygiene was mild to moderate, according to a general oral examination. The radiographs indicated a substantial alveolar bone loss, as well as apical bone loss and a widening of the periodontal ligament space (Fig. 1).

The patient was scheduled for periodontal therapy. Oral hygiene education, scaling, and root planning was all part of the early stages of periodontal treatment. Within a week, the periodontal treatment resulted in considerable improvements in oral hygiene and gum health. The central and lateral incisors were endodontically treated and replanted in single visit. Despite the chance that the tooth may not survive, the patient agreed to reimplantation. The written consent was signed by the patient.

Intentional re-implantation procedure.

After the administration of local anesthesia sulcular incision was made with 15 number blade from distal aspect of maxillary right canine to distal aspect of left canine and full thickness mucoperiosteal flap was reflected both on labial and palatal aspects. Atraumatic extraction of maxillary right central was done. The endodontic therapy was done to the extracted tooth, later scaling and root planing was performed with the ultrasonic scaler tips to remove the calculus and necrotic cementum. On the other hand, the periodontally affected teeth i.e irt 13,12,21,22,23 the granulation tissue was debrided with the surgical curettes and xenogenic bone graft was placed and covered with the collagen membrane, the socket walls were prepared by drilling at a low speed and chilling with sterile saline and the extruded tooth site was then placed into the socket in an adequate position contacting their roots directly to the alveolar bone. Sling sutures were placed irt 13,12,11,21,22,23. The tooth surface was reshaped to prevent any occlusal trauma to occur on the tooth. To stabilize the replanted tooth, the splinting was done with wire and composite on the palatal aspect.

Post operative instructions: Patient was advised not to eat stiff foods using maxillary anteri-or teeth at least for 3 months. Patient was prescribed analgesic and also recommended to use inter-dental brush at the replantation site, in addition to the routine oral hygiene attempts.

**Figure 2:**
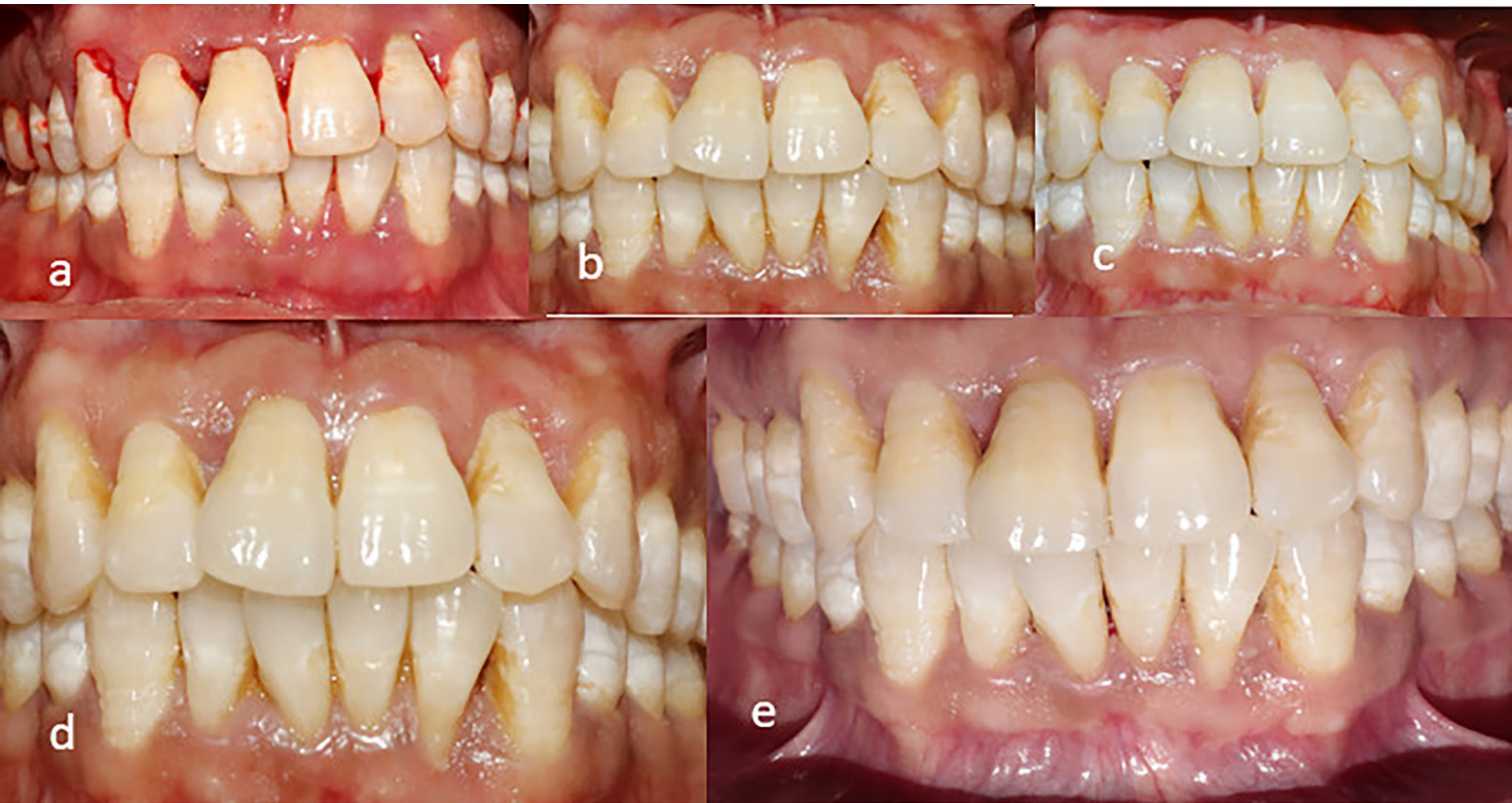
a) pre-operative frontal view b)6 months post-operatively c) 1 year post operatively d) 2 years post operatively e)3 years post operatively.

**Figure 3:**
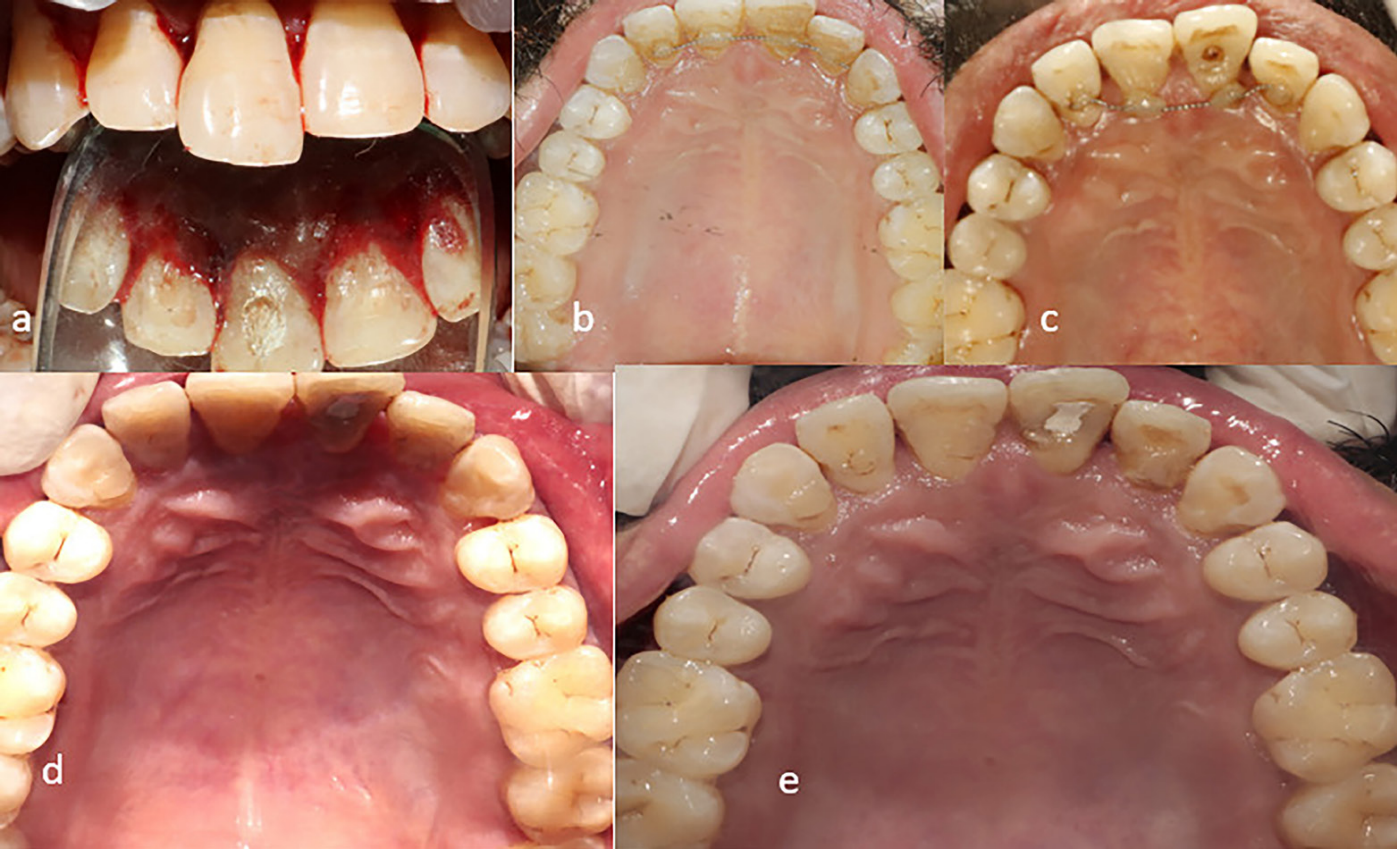
a) pre-operative palatal view b)6 months post-operatively c) 1 year post operatively d) 2 years post operatively e)3 years post operatively.

**Figure 4:**
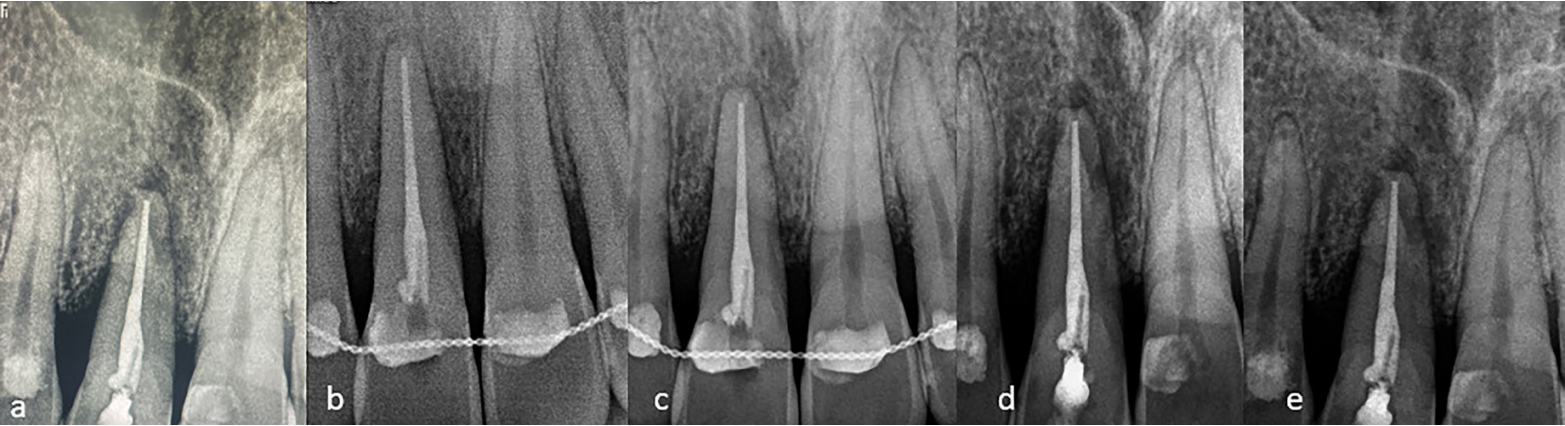
a) Radiographic image depicts- immediately after intentional re-implantation b) Radiograph after 6 months follow up c) Radiograph after 1 year follow up d) Radiograph after 2 years follow up e) Radiograph after 3 years.

Chlorhexidine mouthwash 0.2% was prescribed twice daily for 14 days. Pa-tient was placed in a maintenance recall. The necessary oral prophylaxis was done, oral hygiene in-structions were reinforced and splint integrity was checked along with clinical parameters at every recall visit and in the 2nd year recall visit the splint was removed. In the 3 years recall visit the teeth was stable without any untoward effects and radiographic crestal bone levels shown external root resorption (Figs. 2,3,4).

## Discussion

Patient satisfied with the replantation procedure without any complication and no postoperative discomfort was observed. Clinically, the gingiva around the involved tooth was firm and pink, and there was no bleeding during probing in the patient at the time of examination. Radiograph taken at three years after replantation revealed external root resorption. Some gain in supporting bone tissue around the teeth was determined.

This case report describes the clinical and radiological consequences of the intentional replantation of teeth, which are seen as hopeless due to severe periodontal destruction. In intentionally replanted teeth, the most common causes of failure are external inflammatory resorption or replacement resorption and ankylosis caused by PDL damage and further necrosis of the PDL and cementum (6,11). These complications are related to the degree of PDL damage (11,12). Although no universally accepted protocol has been given for intentional replantation, various techniques and methods have been suggested by different authors (13). Demiralp, *et al*. replanted the desperate teeth periodontally and achieved positive results at the 6th month (1). Yaprak, *et al*. intentional replantation of two central teeth that were mobile due to advanced periodontal disease followed after 4 years (14). In current cases, a similar treatment plan has been made and followed for three years (15). At the end of all procedures, patients are satisfied both aesthetically and functionally.

## Conclusions

For hopeless teeth that are periodontally affected, intentional replantation may be a viable solution. Because they promote healthy gums, a noTable decrease in pocket depth, and the production of new bone, restored teeth hold great promise for maintaining periodontally compromised teeth levels of bone.

## Data Availability

The datasets used and/or analyzed during the current study are available from the corresponding author.
